# π‐Turns in Peptides: A Crystal‐State Literature Survey

**DOI:** 10.1002/psc.70036

**Published:** 2025-06-15

**Authors:** Barbara Biondi, Fernando Formaggio, Claudio Toniolo, Cristina Peggion, Marco Crisma

**Affiliations:** ^1^ CNR – Institute of Biomolecular Chemistry Padova Unit Padova Italy; ^2^ Department of Chemical Sciences University of Padova Padova Italy

**Keywords:** linear and cyclic peptides, peptide conformation, statistical analysis, X‐ray diffraction, π‐turns

## Abstract

The results of an analysis on the presence of π‐turns, characterized by an *i* ← *i* + 5 C=O···H–N intramolecular hydrogen bond, in the X‐ray diffraction structures of peptides are discussed. The survey returned a total of 55 π‐turn occurrences in linear and cyclic peptides. π‐Turns characterized by a helical conformation for residue *i* + 4, but with a screw sense opposite to that of the three preceding residues, are largely prevailing. They are often found at the C‐end of incipient or fully developed α‐helices, 3_10_‐helices, and mixed α‐/3_10_‐helices, thus acting as a C‐capping motif. However, the structures of two linear peptides and 15 cyclopeptides indicate that these types of π‐turns can exist in isolation, without the support of a preceding helix. The frequent presence of additional intramolecular hydrogen bonds internal to the π‐turn is also investigated. Cyclopeptides offered examples of two types of π‐turns that have no parallel in the structures of proteins. Differently from proteins, π‐turns characterized by helical ϕ, ψ sets of the same screw sense for all internal residues are hitherto unreported in the X‐ray diffraction structures of peptides. A suggestion for the rational design in peptides/peptidomimetics of a π‐turn featuring the screw‐sense reversal of residue *i* + 4 is proposed.

## Graphical abstract

The results of an analysis on the presence of π‐turns, characterized by an *i* ← *i* + 5 C=O···H–N intramolecular hydrogen bond, in the X‐ray diffraction structures of peptides are discussed. π‐Turns are often found at the C‐end of incipient or fully developed α‐helices, 3_10_‐helices, and mixed α‐/3_10_‐helices, thus acting as a C‐capping motif.

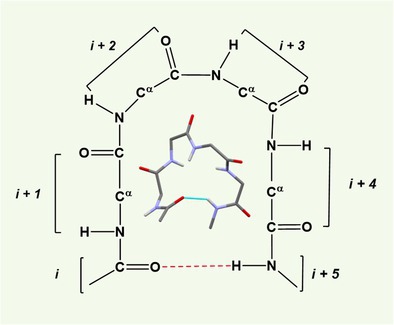

## 1 Introduction

Intramolecularly hydrogen‐bonded turns, in which an N–H···O=C hydrogen bond takes place between nonconsecutive residues, are significant contributors to the 3D architecture of peptides and proteins [1]. In general, they might induce kinks, bends, and even reversal of the direction of the peptide backbone. Of particular relevance are the classes of turns characterized by a hydrogen bond between the C=O group of a given residue and the N–H group of another residue positioned downstream in the peptide chain (assuming for this latter the usual numbering of residues from the N‐terminus to the C‐terminus). They can be schematized as C=O(*i*) ← H–N(*i + n*), where *i* and *i + n* are the residue numbers of the acceptor and the donor, respectively, of the hydrogen bond. The representation can be simplified as *i* ← *i + n*, with the arrow pointing from the hydrogen‐bonding donor to the acceptor. According to this notation, turns can be partitioned into different classes depending on the values of *n*. The number of complete residues encompassed within the turn is *n* − 1. Also, in the case of a peptide chain composed of α‐amino acid residues, the number of atoms in the *pseudo*cycle closed by the hydrogen bond is 3 × (*n* − 1) + 4.

Table 1 summarizes the far from univocal nomenclature that may be found in the literature to classify the most important hydrogen‐bonded turns. Common names based on Greek letters are largely prevailing. They are rooted in the early days of the quest for regular secondary structures of the polypeptide chain. For example, the α‐turn was proposed as the building unit of the α‐helix [2, 3], whereas β‐turns were identified as responsible for the reversal of the chain direction in the antiparallel β‐sheet conformation [4]. Other ways of denoting the various classes of turns, grouped as “Synonyms” in Table 1, are also in use. The “*i* ← *i+n*” notation is explained in the previous paragraph. A similar terminology, based again on the numerical relationships between hydrogen‐bonding donor and acceptor residues, assigns Number 1 to the acceptor. For example, a β‐turn may be indicated as either *i* ← *i +* 3 or 4 → 1 hydrogen‐bonded turn. Finally, in the “C_x_” notation, the capital letter “C” stands for “cyclo,” and the subscript refers to the number of atoms in the *pseudo*‐annular structure closed by the hydrogen bond, thus leading to the term “C_10_ structure” as a synonym for β‐turn.

**TABLE 1:** Nomenclature for turns featuring an intramolecular hydrogen bond between a backbone C=O group and the N–H group of a residue downstream in the peptide chain.

Common name	γ‐turn	β‐turn	α‐turn	π‐turn
Synonyms	*i* ← *i* + 2	*i* ← *i* + 3	*i* ← *i* + 4	*i* ← *i* + 5
	3 → 1	4 → 1	5 → 1	6 → 1
	C_7_	C_10_	C_13_	C_16_
Number of complete amino acid residues internal to the turn	1	2	3	4
Common name of the related helix	γ‐helix	3_10_‐helix	α‐helix	π‐helix
Helix systematic designator	2.2_7_‐helix	3.0_10_‐helix	3.6_13_‐helix	4.4_16_‐helix

The backbone conformation that allows the formation of the intramolecular hydrogen bond is described by the ω, ϕ, and ψ torsion angles internal to the turn. Different combinations of these latter parameters are possible for each class of turns. Within a given *class*, different turn *types* can be assigned to specific conformations or groups of conformations based on some sort of similarity/dissimilarity criteria. Sometimes, in the literature, confusion may be found between turn class and turn type. In this article, we comply with the prevailing terminology that connects turn classes to the hydrogen‐bonding schemes and turn types to the backbone conformations. Interestingly, a turn type characterized by identical ϕ, ψ sets for all residues, if occurring repeatedly and consecutively in the peptide backbone, might give rise to a helix. The common names for such helices mirror those of the corresponding turn classes, with the exception of the 3_10_‐helix [5], which is based on β‐turns (Table 1). A system of naming helices that specifies the number of residues per helical turn (e.g., 3.3 in the case of the α‐helix), followed by a subscript indicating the number of atoms in the *pseudo*cycle closed by the hydrogen bond, is also of use.

An ample body of literature is available, including exhaustive review articles, on peptide β‐turns [6, 7, 8, 9, 10, 11, 12, 13]. Similar contributions about α‐turns [14, 15, 16, 17, 18, 19, 20, 21, 22] and γ‐turns [23, 24, 25, 26, 27, 28, 29, 30, 31] are less numerous. In any case, the occurrences of these two latter classes of turns in the X‐ray diffraction structures of peptides have also been recently reviewed [32, 33].

**FIGURE 1:**
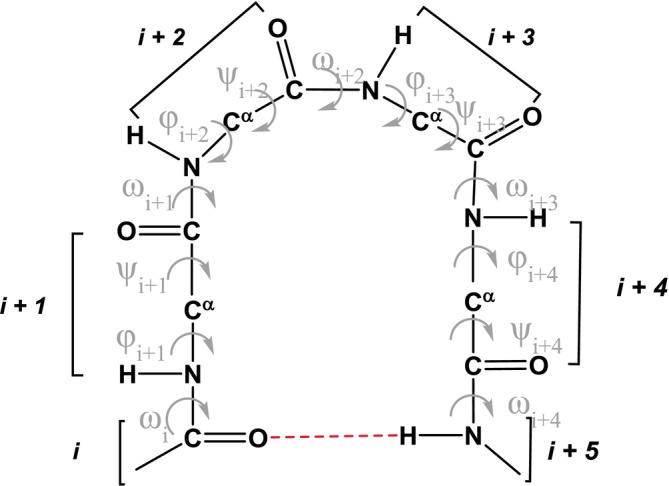
Schematic representation of a π‐turn with numbering of amino acid residues and of relevant backbone torsion angles exploited in this work. The *i* ← *i* + 5 C=O…H–N intramolecular hydrogen bond is indicated by a dashed line.

The π‐turn, focus of this article, is characterized by an *i* ← *i +* 5 intramolecular hydrogen bond, and therefore, it encompasses four amino acid residues (Figure 1). In 1952, a helix based on this hydrogen‐bonding pattern and characterized by 4.4 residues per turn, termed π‐helix, was postulated by Low and Baybutt [34]. This canonical π‐helix has a shorter helical pitch and a larger diameter if compared to the α‐helix proposed by Pauling 1 year earlier [35]. As a consequence, an inner void of about 1 Å in diameter along the π‐helix axis prevents the occurrence of the favorable intra‐helical van der Waals interactions that contribute to the stabilization of the α‐helix [36]. X‐ray diffraction patterns quite consistent with the geometry of the canonical π‐helix were reported for oriented samples of poly(β‐phenethyl L‐aspartate) and poly(β‐phenylpropyl L‐aspartate) [37, 38, 39]. However, until now, only irregular π‐helical stretches have been found in proteins [36, 40, 41, 42]. Sometimes, in α‐helices, the *i* ← *i* + 4 hydrogen‐bonding pattern is interrupted by the insertion of one or a few (π‐turn) *i* ← *i* + 5 hydrogen bonds. These perturbations of the α‐helix regularity (designated in the literature as wide turns, π‐helical regions, or π‐bulges) might be endowed with biological significance [36, 40, 41, 42, 43, 44, 45, 46, 47, 48].

Beside isolated π‐turns, which do also occur in proteins [40, 41], a relevant case is represented by a π‐turn characterized at position *i* + 4 by a residue in a left‐handed helical conformation (i.e., with a screw sense opposite to that of the preceding, right‐handed helical residues), located at the C‐terminus of an α‐helix. Therefore, such π‐turn acts as a helix terminator (C‐cap). It is usually referred to as the Schellman motif (or Schellman loop) [49, 50, 51, 52, 53, 54, 55, 56]. Its *i* ← *i +* 5 intramolecular hydrogen bond is often accompanied by additional hydrogen bonds internal to the π‐turn. The Schellman motif was reported to be found in about 25% of helices in globular proteins [50, 55, 56].

Overall, π‐turns in proteins have been extensively and in depth investigated. Conversely, much more limited information may be found in the secondary literature about the presence of π‐turns in the X‐ray diffraction structures of peptides. Indeed, apart from a few examples of structures containing a π‐turn described in review articles that are not exclusively focused on this topic [57, 58, 59], to the best of our knowledge, the only comprehensive report may be found in the analysis published by Rajashankar and Ramakumar in 1996 about π‐turns in proteins and peptides [40]. They listed nine structures of peptides containing a π‐turn. After almost three decades, the aim of the present contribution is to provide an update of that work. Specifically, we herewith report an in‐depth analysis of the π‐turns observed in linear and cyclic peptides, the crystal‐state structures of which are currently available, in order to provide a better understanding of this class of intramolecularly hydrogen‐bonded turns. It is worth recalling that the level of detail offered by X‐ray diffraction of peptides, at atomic resolution, is not routinely achievable by macromolecular crystallography of proteins.

## 2 Methods

A search was performed on the Cambridge Structural Database (CSD, release 2024.3) [60] for structures containing the fragment ‐C(=O)‐[N‐C‐C(=O)]_4_‐NH‐ in which the NH_*i* + 5_ and C_*i*_ = O_*i*_ groups are involved in a C_16_ structure, based on the intramolecular H‐bonding criteria that the O_*i*_···H_*i* + 5_ distance must be within 2.50 Å (i.e., less than the sum of van der Waals radii of H and O) and the N_*i* + 5_–H_*i* + 5_···O_*i*_ angle ≥ 120° [61, 62, 63]. To this aim, the ConQuest [64] software package was exploited, which inter alia allowed retrieval of the values of relevant backbone torsion angles. Considering that in less recent years, it quite often happened that X‐ray diffraction structures were deposited in the CSD without coordinates of H atoms, a second search was performed for the fragment ‐C(=O)‐[N‐C‐C(=O)]_4_‐N‐ with the O_*i*_···N_*i* + 5_ distance less than 3.30 Å as the fundamental criterion. After removal of the entries already identified through the first search, the remaining entries, potentially containing a C_16_ structure, were critically evaluated through analysis with the aid of the program Mercury [65] and comparison with the H‐bonding schemes reported in the original publications.

We excluded from our investigation peptide molecules containing β‐ or γ‐amino acids, or α‐hydroxy acids, or modifications of the peptide linkage between two α‐amino acids that give rise to the so‐called *pseudo*peptides. An exception to these criteria was made in the case of a couple of structures that, in our opinion, are of particular relevance. Also, we omitted structures in which the direct H‐bond between the C=O group of residue *i* and the N–H group of residue *i* + 5 is replaced by an indirect interaction mediated by the insertion of a water molecule. An example of this latter arrangement may be found in [66].

As for cyclic peptides, we took into consideration only molecules in which the cycle is entirely constituted of α‐amino acids. In other words, molecules in which the cyclic skeleton either combines peptide and nonpeptide portions or results from covalent bonding between two side chains (as well as from side‐chain to backbone cyclization) of an otherwise linear peptide backbone were not retained.

The methodology described previously parallels that exploited in our previous survey on the occurrence of isolated α‐turns in the X‐ray diffraction structures of peptides [32].

## 3 Results and Discussion

The results of our survey on the occurrence of π‐turns in the X‐ray diffraction structures of peptides are given in Tables 2 and 3 for linear and cyclic peptides, respectively. For each entry, the four residues encompassed within the turn (*i* + 1 to *i* + 4) are underlined. The values of backbone torsion angles internal to the 16‐membered H‐bonded *pseudo*cycle are reported, accompanied by the available intramolecular H‐bond parameters. We found 38 examples of π‐turns in 31 structures of linear peptides [67, 68, 69, 70, 71, 72, 73, 74, 75, 76, 77, 78, 79, 80, 81, 82, 83, 84, 85, 86, 87, 88], ranging in length from five to 17 residues, and 17 examples in 15 structures of cycloocta‐ and cyclononapeptides [89, 90, 91, 92, 93, 94, 95, 96, 97, 98, 99, 100, 101, 102].

**TABLE 2:** Relevant backbone torsion angles (°) and intramolecular H‐bond parameters (Å,°) for π‐turns in linear peptides.

Entry	ω_*i*_	ϕ_*i* + 1_	ψ_*i* + 1_	ω_*i* + 1_	ϕ_*i* + 2_	ψ_*i* + 2_	ω_*i* + 2_	ϕ_*i* + 3_	ψ_*i* + 3_	ω_*i* + 3_	ϕ_*i* + 4_	ψ_*i* + 4_	ω_*i* + 4_	N_*i* + 5_…O_*i*_	H_*i* + 5_…O_*i*_	N_*i* + 5_–H_*i* + 5_ …O_*i*_	Notes	References
1	Ac‐Aib‐L‐Leu‐L‐Ala‐L‐Leu‐NH‐CH (CH_2_‐Ph)‐CH_2_‐O‐CH_2_‐CO‐L‐Leu‐L‐Ala‐L‐Leu‐Aib‐NH_2_ trihydrate																	[67]
	−179	−64	−22	175	−60	−20	177	−121	35	172	67	19	175	2.957	2.15	155	a,b	
2	Z‐L‐Leu‐Aib‐Gly‐L‐Ile‐L‐Leu‐OMe																	[68]
	−175	42	52	172	59	30	177	103	−1	178	−78	−34	−178	2.958	2.01	171	a,b	
3	Boc‐L‐Leu‐L‐Leu‐Aib‐D‐Leu‐L‐Leu‐Aib‐OMe																	[69]
	175	54	46	175	55	28	175	103	−9	179	−67	−29	180	3.299	2.46	162	a,b	
4	Boc‐L‐Val‐Aib‐L‐Phe‐Aib‐L‐Ala‐Aib‐L‐Leu‐OMe																	[70]
	−171	−79	−38	175	−59	−30	−174	−100	10	172	74	18	172	2.922	2.18	140	b	
5	Boc‐L‐Val‐Aib‐L‐Leu‐Aib‐L‐Ala‐Aib‐L‐Phe‐OMe																	[70]
	−176	−77	−38	179	−55	−37	−175	−87	−5	−172	61	43	180	2.924	2.07	159	b	
6	Boc‐L‐Val‐Aib‐L‐Leu‐Aib‐L‐Ala‐Aib‐L‐Leu‐OMe (two independent molecules, A and B)																	[70]
6A	−178	−58	−30	−176	−56	−32	−174	−113	12	−177	55	42	175	3.080	2.19	169	a,b	
6B	−177	−80	−39	176	−56	−30	−176	−90	−1	−176	62	33	173	2.951	2.19	143	b	
7	Boc‐L‐Leu‐Aib‐L‐Val‐Gly‐L‐Leu‐Aib‐L‐Val‐OMe																	[71]
	−175	−64	−35	177	−70	−19	−179	−106	16	169	67	34	168	3.194	2.34	170	a	
8	Boc‐L‐Leu‐Aib‐L‐Val‐L‐Ala‐L‐Leu‐Aib‐L‐Val‐OMe																	[72]
	−173	−65	−29	178	−70	−25	−175	−109	13	172	67	36	176	3.065	2.00	167	a,b	
9	Boc‐L‐Val‐ΔPhe‐L‐Leu‐L‐Phe‐L‐Ala‐ΔPhe‐L‐Leu‐OMe																	[73]
	176	−75	−40	173	−63	−23	180	−84	−9	−179	96	−4	−178	3.064	2.33	142	b	
10	*N*‐(2‐hydroxy‐4,4‐dimethyl‐6‐oxocyclohex‐1‐ene‐1‐carbonothioyl)‐Gly‐L‐Leu‐Aib‐L‐Val‐L‐Ala‐L‐Leu‐Aib‐L‐Val‐OMe methanol solvate monohydrate																	[74]
	−178	−72	−41	175	−54	−30	−175	−104	8	177	57	44	174	2.993	2.12	170	b	
11	Boc‐ Gly‐L‐Leu‐Aib‐L‐Val‐L‐Ala‐L‐Leu‐Aib‐L‐Val‐OMe																	[74]
	−177	−80	−33	175	−60	−27	179	−98	−6	−174	47	49	−174	3.220	2.34	175	b	
12	*N*,*N*‐[(4,4‐dimethyl‐2,6‐dioxocyclohexylidene)methylene]bis (Gly‐L‐Leu‐Aib‐L‐Val‐L‐Ala‐L‐Leu‐Aib‐L‐Val‐OMe) isopropanol solvate monohydrate (two independent molecules, A and B, each bearing two peptide chains, a and b)																	[74]
12Aa	−178	−66	−41	179	−59	−29	179	−94	14	176	60	35	169	3.080	2.25	163	b	
12Ab	179	−64	−42	178	−60	−28	179	−95	3	178	56	44	173	3.175	2.32	176	b	
12Ba	179	−65	−41	178	−60	−30	−179	−92	7	175	60	41	165	3.227	2.38	166	b	
12Bb	178	−65	−43	179	−58	−29	180	−93	2	179	58	39	171	3.233	2.39	168	b	
13	Boc‐L‐Leu‐Aib‐L‐Glu‐L‐Leu‐L‐Leu‐L‐Ala(3‐pyridyl)‐Aib‐L‐Leu‐OEt acetonitrile solvate																	[75]
	177	−66	−21	175	−62	−23	179	−102	2	−173	51	41	171	3.402	2.47	166	a,b	
14	Ac‐ΔPhe‐L‐Val‐ΔPhe‐L‐Phe‐L‐Ala‐L‐Val‐ΔPhe‐Gly‐OMe acetone solvate dihydrate																	[76]
	180	−78	−8	172	−93	−14	−170	−123	19	179	65	33	169	2.949	2.00	156	a	
15	Boc‐L‐Val‐ΔPhe‐L‐Phe‐L‐Ala‐L‐Leu‐L‐Ala‐ΔPhe‐L‐Leu‐OH monohydrate																	[77]
	174	−64	−23	175	−60	−30	177	−98	15	175	84	10	−170	3.127	2.17	157	a,b	
16	Boc‐L‐Leu‐L‐Leu‐Aib‐L‐Leu‐L‐Leu‐Aib ‐L‐Leu‐D‐Leu‐Aib‐OMe *N*,*N*‐dimethylacetamide solvate																	[78]
	173	−57	−44	179	−56	−30	−178	−96	11	173	92	12	174	3.012	2.16	163	a,b	
17	Boc‐L‐Leu‐D‐Leu‐Aib‐L‐Leu‐L‐Leu‐Aib‐L‐Leu‐D‐Leu‐Aib‐OMe acetonitrile methanol solvate (two independent molecules, A and B)																	[78]
17A	178	−66	−44	−179	−55	−35	−177	−88	−8	−170	56	39	175	2.932	2.08	163	b	
17B	−177	−70	−40	179	−55	−35	−177	−88	−9	−171	66	25	170	2.947	2.21	141	b	
18	Boc‐L‐Leu‐D‐Leu‐Aib‐L‐Leu‐D‐Leu‐Aib‐L‐Leu‐D‐Leu‐Aib‐OMe monohydrate																	[79]
	−176	−58	−46	−173	−57	−34	−175	−92	−1	−173	65	39	178	3.018	2.18	165	b	
19	Boc‐L‐Leu‐L‐Leu‐Aib‐D‐Leu‐L‐Leu‐Aib‐D‐Leu‐L‐Leu‐Aib‐OMe hemihydrate (two independent molecules, A and B)																	[80]
19A	−174	51	57	176	61	35	174	82	1	−169	−99	−11	178	3.056	2.22	160	b	
19B	−170	50	52	177	59	31	−179	86	0	−175	−81	−30	−172	3.273	2.42	168	a,b	
20	Boc‐L‐Leu‐Aib‐L‐Val‐Gly‐L‐Leu‐Aib‐L‐Val‐D‐Ala‐D‐Leu‐Aib‐OMe monohydrate																	[81]
	−177	−65	−39	180	−56	−39	−173	−97	−8	−177	85	31	168	2.803	2.01	153		
21	Boc‐L‐Leu‐Aib‐L‐Val‐D‐Ala‐L‐Leu‐Aib‐L‐Val‐D‐Ala‐D‐Leu‐Aib‐OMe trihydrate (two independent molecules, A and B)																	[81]
21A	−177	−66	−44	−175	−55	−33	−177	−106	24	165	77	21	178	2.911	2.08	164	b	
21B	−177	−63	−39	−177	−58	−30	−179	−112	28	164	68	30	177	3.009	2.17	167	b	
22	Boc‐L‐Leu‐Aib‐L‐Val‐Aib‐L‐Leu‐Aib‐L‐Val‐D‐Ala‐D‐Leu‐Aib‐OMe monohydrate																	[81]
	174	−81	−45	−176	−53	−41	−173	−102	7	173	52	43	−179	3.009	2.19	157	b	
23	Boc‐L‐Leu‐Aib‐L‐Val‐L‐Ala‐L‐Leu‐Aib‐L‐Val‐D‐Ala‐D‐Leu‐Aib‐OMe																	[82]
	179	−68	−44	−172	−59	−45	−169	−106	−7	−179	84	42	176	2.938	2.15	153	c	
24	*p*BrBz‐Aib‐(L‐Ala‐Aib)_2_‐L‐Ala‐Aib‐ L‐Ala‐Aib‐L‐Ala‐OMe tetrahydrate																	[83]
	−174	−72	−39	177	−57	−33	−177	−96	16	168	68	31	166	3.052	n.a.	n.a.	a,b	
25	*p*BrBz‐Aib‐(L‐Ala‐Aib)_2_‐L‐Ala‐Aib‐ L‐Ala‐Aib‐L‐Ala‐OMe dimethylsulfoxide solvate																	[84]
	−178	−68	−35	178	−54	−35	−176	−93	10	−179	62	39	179	3.077	n.a.	n.a.	a,b	
26	Boc‐(L‐Ala‐Aib)_2_‐L‐Glu (OBzl)‐L‐Ala‐Aib‐L‐Ala‐Aib‐L‐Ala‐OMe dichloromethane solvate																	[85]
	179	−69	−47	180	−56	−36	−175	−79	−22	−164	56	46	−178	3.073	n.a.	n.a.	b	
27	*p*BrBz‐Aib‐(L‐Ala‐Aib)_3_‐L‐Ala‐Aib‐ L‐Ala‐Aib‐L‐Ala‐OMe dihydrate																	[83]
	−177	−72	−37	178	−58	−29	180	−92	7	−179	66	28	177	2.977			b	
28	Boc‐(L‐Leu‐D‐Leu‐Aib)_2_‐L‐Leu‐D‐Leu‐Aib‐L‐Leu‐D‐Leu‐Aib‐OMe 1,4‐dioxane solvate dihydrate																	[79]
	179	−57	−51	−178	−58	−35	−178	−73	−18	−172	85	16	−175	2.987	2.16	157	b	
29	Boc‐(L‐Leu)_2_‐Aib‐D‐Leu‐D‐Leu‐Aib‐D‐Leu‐D‐Leu‐Aib‐(L‐Leu)_2_‐ Aib‐OMe butan‐2‐one solvate																	[86]
	−178	71	46	−176	55	37	178	87	−5	−172	−61	−32	179	2.979	2.12	166	b	
30	Boc‐L‐Val‐L‐Ala‐L‐Leu‐Aib‐L‐Val‐L‐Ala‐L‐Leu‐L‐Val‐L‐Ala‐L‐Leu‐Aib‐L‐Val‐L‐Lac‐L‐Leu‐OMe cyclopentane solvate																	[87]
	179	−70	−45	−179	−58	−40	−174	−89	4	−177	55	36	167	2.967	2.19	144	b	
31	Boc‐L‐Val‐L‐Ala‐L‐Leu‐Aib‐L‐Val‐L‐Ala‐L‐Leu‐Gly‐Gly‐L‐Leu‐L‐Phe‐L‐Val‐D‐Pro‐Gly‐L‐Leu‐L‐Phe‐L‐Val‐OMe dihydrate																	[88]
	−176	−71	−39	178	−60	−32	−176	−97	16	171	93	11	−178	2.818	2.02	147	b	

*Note:* The amino acid sequence within the π‐turn is underlined. a, β‐turn (*i* ← *i* + 3) within the π‐turn; b, β‐turn (*i* + 1 ← *i* + 4) within the π‐turn; and c, α‐turn (*i* ← *i* + 4) within the π‐turn.

Abbreviations: Aib, α‐aminoisobutyric acid; Lac, lactic acid; n.a., data not available; ΔPhe, C^α^,C^β^‐didehydrophenylalanine.

**TABLE 3:** Relevant backbone torsion angles (°) and intramolecular H‐bond parameters (Å,°) for π‐turns in cyclic peptides.

Entry	ω_*i*_	ϕ_*i* + 1_	ψ_*i* + 1_	ω_*i* + 1_	ϕ_*i* + 2_	ψ_*i* + 2_	ω_*i* + 2_	ϕ_*i* + 3_	ψ_*i* + 3_	ω_*i* + 3_	ϕ_*i* + 4_	ψ_*i* + 4_	ω_*i* + 4_	N_*i* + 5_…O_*i*_	H_*i* + 5_…O_*i*_	N_*i* + 5_–H_*i* + 5_ …O_i_	Notes	References
1	c‐(L‐Leu‐D‐Phe‐L‐Pro‐L‐Pro‐L‐MeAla‐D‐Ile‐D‐Pro‐D‐MeLeu) 2‐propanol solvate hydrate																	[89]
	170	89	−124	4	−67	161	−6	−81	160	11	−124	20	174	3.189	2.33	164		
2	Squamtin A; c‐(L‐Ala‐L‐Ile‐L‐Pro‐L‐Met(O)‐L‐Tyr‐Gly‐L‐Thr‐L‐Val) (two, differently hydrated, pseudopolymorphic structures, A and B)																	[90]
2A	−174	−66	−22	176	−63	−16	174	−122	20	171	85	19	176	3.191	2.34	169	a,b	
2B	−174	−65	−22	177	−64	−16	173	−120	21	171	82	21	174	3.114	n.a.	n.a.	a,b	
3	c‐(L‐Leu‐L‐Pro‐L‐MeLeu‐L‐Ala‐L‐MeLeu‐L‐Pro‐L‐Leu‐D‐Ala) hydrate																	[89]
	−174	−59	−35	−178	−66	−12	176	−104	6	180	70	33	−178	3.279	2.42	164	a,b	
4	c‐(L‐Pro‐L‐Pro‐L‐Phe‐L‐Phe‐Ac_6_ c‐L‐Ile‐D‐Ala‐L‐Val) dimethyl sulfoxide solvate trihydrate (two independent molecules, A and B)																	[91]
4A	178	−58	−31	−178	−57	−24	179	−113	3	177	93	4	−174	3.199	2.42	151	a,b	
4B	−171	−58	−39	−173	−62	−26	−178	−103	4	175	97	7	−173	2.263	2.45	159	a,b	
5	Ile‐3‐S‐deoxo‐amaninamide; bicyclo‐(L‐Asn‐L‐Hyp‐L‐Ile‐L‐Trp*‐Gly‐L‐Ile‐Gly‐L‐Cys*) hydrate																c	[92]
	176	−75	130	−178	121	−22	177	−127	14	176	132	−1	180	2.828	2.10	139		
6	Cyclolinopeptide A 2‐propanol solvate monohydrate; c‐(L‐Pro‐L‐Pro‐L‐Phe‐L‐Phe‐L‐Leu‐L‐Ile‐L‐Ile‐L‐Leu‐L‐Val)																	[93]
	180	−61	−28	179	−55	−33	179	−115	22	176	55	48	169	3.013	n.a.	n.a.	a,b	
7	Cyclolinopeptide A acetonitrile solvate; c‐(L‐Pro‐L‐Pro‐L‐Phe‐L‐Phe‐L‐Leu‐L‐Ile‐L‐Ile‐L‐Leu‐L‐Val) acetonitrile solvate																	[94]
	−174	−65	−27	176	−51	−34	179	−121	27	174	54	48	175	3.059	2.28	157	a,b	
8	Cyclolinopeptide A dimethylsulfoxide solvate; c‐(L‐Pro‐L‐Pro‐L‐Phe‐L‐Phe‐L‐Leu‐L‐Ile‐L‐Ile‐L‐Leu‐L‐Val) dimethylsulfoxide solvate																	[95]
	180	−60	−34	179	−53	−29	180	−124	24	176	59	40	173	2.970	2.12	162	a,b	
9	Cyclolinopeptide A methanol solvate; c‐(L‐Pro‐L‐Pro‐L‐Phe‐L‐Phe‐L‐Leu‐L‐Ile‐L‐Ile‐L‐Leu‐L‐Val) methanol solvate																	[96]
	−179	−63	−34	−177	−57	−31	179	−113	22	175	58	41	173	2.975	2.13	161	a,b	
10	c‐(L‐Pro‐L‐Pro‐L‐Phe‐L‐Phe‐Aib‐Aib‐L‐Ile‐D‐Ala‐L‐Val) methanol solvate dihydrate																	[97]
	−179	−54	−41	−173	−55	−27	−166	−123	4	172	77	28	179	3.118	n.a.	n.a.	a,b	
11	c‐(L‐Pro‐L‐Pro‐L‐Phe‐L‐Phe‐Aib‐Aib‐L‐Ile‐D‐Ala‐L‐Val) acetonitrile solvate dihydrate																	[98]
	−178	−54	−42	−174	−53	−33	−174	−117	16	172	78	27	179	3.086	2.15	165	a,b	
12	Cyclolinopeptide B methanol solvate; c‐(L‐Pro‐L‐Pro‐L‐Phe‐L‐Phe‐L‐Val‐L‐Ile‐L‐Met‐L‐Leu‐L‐Ile) methanol trisolvate																	[99]
	−163	−64	−48	−177	−70	−20	175	−83	−4	−172	53	43	173	3.170	2.26	168	a,b	
13	c‐(L‐Ala‐L‐Leu‐L‐Leu‐L‐Leu‐L‐Val‐L‐Leu‐L‐Val‐L‐Leu‐L‐Pro) monohydrate																	[100, 101]
	−171	−69	−24	174	−51	−36	−178	−114	23	175	53	45	174	3.122	2.15	166	a,b	
14	c‐(L‐Ile‐L‐Leu‐L‐Leu‐L‐Leu‐L‐Val‐L‐Leu‐L‐Val‐L‐Leu‐L‐Pro) monohydrate																	[100]
	−163	−75	−23	175	−61	−24	178	−113	20	177	54	43	174	3.130	2.28	164	a,b	
15	c‐(L‐Ile‐L‐Leu‐L‐Leu‐L‐Leu‐L‐Val‐D‐Leu‐L‐Val‐L‐Leu‐L‐Pro) methanol solvate monohydrate																	[102]
	−171	−67	−29	175	−48	−39	−173	−113	17	173	66	38	177	3.170	2.31	165	a,b	

*Note:* The amino acid sequence within the π‐turn is underlined. a, β‐turn (*i* ← *i* + 3) within the π‐turn; b, β‐turn (*i* + 1 ← *i* + 4) within the π‐turn; and c, cyclization between side chains of the starred residues.

Abbreviations: Ac_6_c, 1‐aminocyclohexane carboxylic acid; Aib, α‐aminoisobutyric acid; MeAla, *N*‐methylalanine; MeLeu, *N*‐methylleucine; Met(O), methionine sulfoxide; n.a., data not available.

The N‐terminal residue of the linear peptides listed in Table 2 is either acylated or urethane protected, apart from Entry 10, featuring a thioamide at the N‐terminus, and Entry 12, in which two peptide chains are connected to a cyclohexylidene moiety. The C‐terminal residue is esterified, except in the cases of Entry 1, terminating with a primary amide, and Entry 15, bearing a free carboxyl group. A *pseudo*peptide (Entry 1) and a depsipeptide (Entry 30) are included in Table 2. In Entry 1, the peptide bond between the residue that provides the H‐bond acceptor for the π‐turn and the preceding residue is replaced by a ‐CH_2_‐O‐ moiety. Entry 30 contains an ester bond between the carbonyl group of residue *i* + 5 (acting as the H‐bond donor for the π‐turn) and the following hydroxy acid residue.

Among the 55 occurrences of π‐turns, there is only one example featuring the *cis‐*disposition for some of the ω torsion angles within the turn. Specifically, in the X‐ray diffraction structure of the cyclic octapeptide c‐(L‐Leu‐D‐Phe‐L‐Pro‐L‐Pro‐L‐MeAla‐D‐Ile‐D‐Pro‐D‐MeLeu), Entry 1 of Table 3, the *cis‐*disposition is observed for the tertiary peptide bonds between D‐Phe (2) and L‐Pro (3) (ω_*i* + 1_ = 4°), between L‐Pro (3) and L‐Pro (4) (ω_*i* + 2_ = −6°), and between L‐Pro (4) and L‐MeAla (5) (ω_*i* + 3_ = 11°). Among the cyclopeptides listed in Table 3, there are two additional compounds, Entries 2 and 3, in which either one or two tertiary peptide bonds occur within the π‐turn. In these latter cases, however, the disposition of all ω torsion angles internal to the π‐turn is *trans*, similarly to all other entries of Tables 2 and 3. In general, for both linear and cyclic peptides listed in Tables 2 and 3, deviations of ω torsion angles from the exact *trans*‐planarity (180°) do not exceed ±10°. Among the linear peptides (Table 2), slightly larger deviations, in the range of 11°–16°, are found for ω_*i* + 2_ of Entry 23, ω_*i* + 3_ of Entries 7, 19A, 21A, 21B, 24, and 26, and ω_*i* + 4_ of Entries 7, 12Aa, 12Ba, 14, 20, 24, and 30. Similarly, in the case of cyclic peptides (Table 3), deviations of ω torsion angles from the exact *trans*‐planarity larger in magnitude than 10° and up to 17° are found only in a few cases, namely, ω_*i*_ of Entries 12 and 14, ω_*i* + 2_ of Entry 10, and ω_*i* + 4_ of Entry 6.

### 3.1 π‐Turn Classification

**FIGURE 2:**
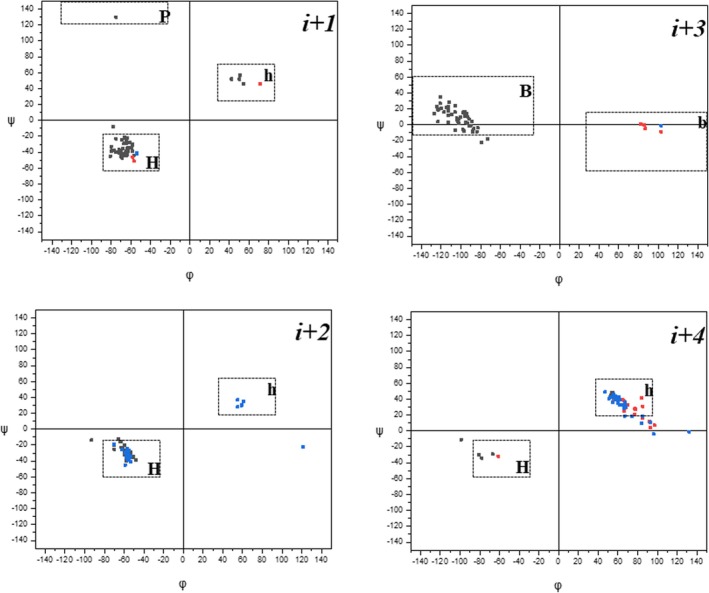
Distribution of ϕ,ψ torsion angles for residues at positions *i* + 1, *i* + 2, *i* + 3, and *i* + 4 internal to π‐turns in the X‐ray diffraction structures of peptides. Residues of L configuration are in black, D configuration in red, and achiral configuration in blue.

The distributions of the ϕ,ψ sets of residues occupying positions *i* + 1, *i* + 2, *i* + 3, and *i* + 4, respectively, internal to each π‐turn for both linear and cyclic peptides characterized by the *trans‐*disposition for all ω torsion angles within the turn (i.e., except Entry 1 of Table 3) are illustrated in Figure 2, Panels A–D. Each panel contains 54 datapoints, many of which overlap. Interestingly, at position *i* + 1, there are 49 residues of L configuration (45 of which are characterized by a negative value of ϕ and four by a positive value of ϕ), with only three examples of D configuration (two with ϕ negative and one positive) and two achiral (both with ϕ negative). Conversely, at position *i* + 2, the populations of configurations are 25 L (all with ϕ negative), 29 achiral (23 of which with ϕ negative and six positive), and none D. At position *i* + 3, we found 49 L residues (all with ϕ negative), four D residues (all with ϕ positive), and one achiral residue (ϕ positive). At position *i* + 4, there are only 11 L residues (four of which with ϕ negative and seven positive), 16 D residues (all with ϕ positive), and as many as 26 achiral residues (all characterized by a positive value of ϕ). Overall, it appears that achiral residues predominantly mimic an L residue when in position *i* + 2 but a D residue when occupying position *i* + 3.

To classify the conformation of each residue, we follow an approach similar to that exploited by us in our previous analysis on the occurrence of intramolecularly H‐bonded, isolated α‐turns in the X‐ray diffraction structures of peptides [32]. Specifically, we describe the conformation of each residue in our dataset as belonging to one of the following areas of the Ramachandran map:

- “H” (right‐handed helical), −90° ≤ ϕ ≤ −30°, −65° ≤ ψ ≤ −15°;
- “B” (bridge, ϕ negative), −150° ≤ ϕ ≤ −30°, −15° ≤ ψ ≤ 60°;
- “P” (polyPro like, ϕ negative), −130° ≤ ϕ ≤ −30°, 120° ≤ ψ ≤ 180°;
- “h” (left‐handed helical), 30° ≤ ϕ ≤ 90°, 15° ≤ ψ ≤ 65°;
- “b” (bridge, ϕ positive), 30° ≤ ϕ ≤ 150°, −60° ≤ ψ ≤ 15°; and
- “p” (polyPro like, ϕ positive), 30° ≤ ϕ ≤ 130°, −180° ≤ ψ ≤ −120°.

Then, for each π‐turn, a conformational descriptor is built through the combination of the four labels assigned to residues *i* + 1, *i* + 2, *i* + 3, and *i* + 4.

### 3.2 Type HHBh

From Figure 2, it appears that the most populated regions of the ϕ,ψ space are “H” for positions *i* + 1 and *i* + 2, “B” for position *i* + 3, and “h” for *i* + 4. Not surprisingly, therefore, the “HHBh” conformational descriptor accounts for about 75% of all π‐turns for both linear peptides (Table 2: Entries 1, 4–8, 10–13, 15, 17A–18, 20–25, 27, and 30) and cyclopeptides (Table 3: Entries 2A, 2B, and 6–15). Within this group of entries, the ϕ_*i* + 1_ values range from −81° to −54°, and the ψ_*i* + 1_ values range from −46° to −21°. At position *i* + 2, the spread of ϕ values slightly decreases and that of ψ values increases (ϕ_*i* + 2_ from −70° to −51° and ψ_*i* + 2_ from −45° to −16°). The ϕ,ψ values for positions *i* + 1 and *i* + 2 as averaged from the 27 occurrences of the HHBh π‐turn conformation in linear peptides are ϕ_*i* + 1_,ψ_*i* + 1_ = −68°,–38° and ϕ_*i* + 2_,ψ_*i* + 2_ = −58°,–31°, respectively. Those averaged from the 12 occurrences in cyclopeptides are very similar (ϕ_*i* + 1_,ψ_*i* + 1_ = −63°,–31°; ϕ_*i* + 2_,ψ_*i* + 2_ = −57°,–28°). The “bridge” conformation of residue *i* + 3 is characterized by ϕ_*i* + 3_ values ranging from −124° to −83° and ψ_*i* + 3_ from −8° to 35°. The average ϕ,ψ values for residue *i* + 3 of linear peptides (ϕ_*i* + 3_,ψ_*i* + 3_ = −99°, 7°) differ quite significantly from those averaged from cyclic peptides (ϕ_*i* + 3_,ψ_*i* + 3_ = −115°, 18°). Position *i* + 4 is characterized by a left‐handed helical conformation (“h” in our notation), that is, by a screw‐sense reversal with respect to the preceding residues. The ϕ_*i* + 4_ values range from 51° to 85°, and the ψ_*i* + 4_ values range from 10° to 49°. The average ϕ,ψ values for residue *i* + 4 of linear peptides (ϕ_*i* + 4_,ψ_*i* + 4_ = 64°, 35°) and those averaged from cyclic peptides (ϕ_*i* + 4_,ψ_*i* + 4_ = 64°, 37°) compare well.

In the π‐turns of type HHBh, the right‐handed helical conformation adopted by both residues *i* + 1 and *i* + 2 offers the possibility of the occurrence of a Type‐III β‐turn encompassed within the π‐turn (with the O_*i*_ carbonyl oxygen acting as a double acceptor of H‐bond, from both the N_*i* + 3_–H_*i* + 3_ and N_*i* + 5_–H_*i* + 5_ groups), provided that the ϕ,ψ values of both residues *i* + 1 and *i* + 2 in the “H” region would be sufficiently close to the standard for such folded conformation (ϕ_*i* + 1_,ψ_*i* + 1_ = ϕ_*i* + 2_,ψ_*i* + 2_ = −60°,–30°) [6]. Similarly, the succession of conformations “H” and “B” for residues *i* + 2 and *i* + 3 may allow the onset of a Type‐I β‐turn (with the N_*i* + 4_–H_*i* + 4_ group H‐bonded to the O_*i* + 1_ carbonyl oxygen atom) when the ϕ,ψ sets of residues *i* + 2 and *i* + 3 are not far from −60°, −30° to −90°, 0°, respectively [6].

**FIGURE 3:**
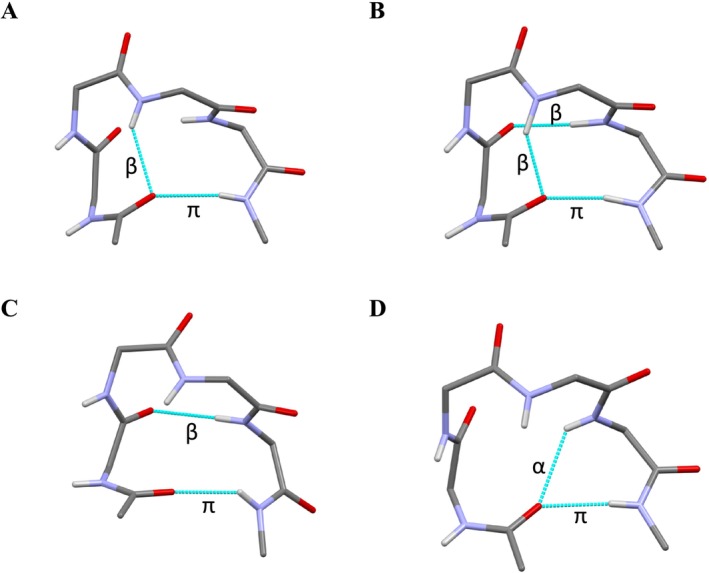
Examples of π‐turns of type HHBh featuring additional intramolecular H‐bonds internally to the π‐turn: (A) β‐turn *i* ← *i* + 3; (B) β‐turn *i* + 1 ← *i* + 4; (C) β‐turns *i* ← *i* + 3 and *i* + 1 ← *i* + 4; and (D) α‐turn *i* ← *i* + 4. Side chains are omitted for clarity.

In both Tables 2 and 3, the occurrence of an intramolecularly H‐bonded β‐turn (C_10_ structure) with residues *i* + 1 and *i* + 2 in the corner positions is indicated with note “a,” while that with residues *i* + 2 and *i* + 3 in the corner positions is indicated with note “b.” Among the 27 occurrences of π‐turns of type HHBh in linear peptides (Table 2), as many as 16 examples (Entries 5, 6B, 10–12Bb, 17A–18, 21A–22, 27, and 30) are found to contain a Type‐I β‐turn encompassing residues *i* + 2 and *i* + 3 (Figure 3B); one example (Entry 7) features a Type‐III β‐turn at the level of residues *i* + 1 and *i* + 2 (Figure 3A), and eight examples (Entries 1, 4, 6A, 8, 13, 15, 24, and 25) contain two consecutive β‐turns within the π‐turn (Figure 3C). On the other hand, all of the 12 examples of π‐turns of type HHBh in cyclic peptides (Table 3) show the presence of two consecutive β‐turns within the π‐turn. We found only one example (Table 2, Entry 23) of the occurrence of an α‐turn (C_13_ structure) within the π‐turn, with the H‐bond between the N_*i* + 4_–H_*i* + 4_ group and the O_*i*_ carbonyl oxygen atom (the latter acting as a double acceptor of the H‐bond) (Figure 3D).

Overall, the occurrence of one or two additional H‐bonds within the π‐turn is almost invariably associated with the conformational descriptor HHBh. Indeed, among the 39 occurrences of the latter (combining linear and cyclic peptides), as many as 18 (46%) contain a single H‐bond of the C_10_ or C_13_ type, and 20 (51%) contain two consecutive C_10_ structures. We found only one example of type HHBh π‐turn lacking additional H‐bonds within the turn. Specifically, in the case of Entry 20 of Table 2, the H···O separations for the two potential *i* ← *i* + 3 and *i* + 1 ← *i* + 4 H‐bonds are 2.64 and 2.55 Å, respectively. The latter value is only slightly above the 2.50 Å upper limit commonly accepted for the occurrence of a C=O···H–N hydrogen bond, and the related N–H···O angle is 148°.

### 3.3 Types HHBb, HHHh, HBBh, and BBBh

Entries 9, 16, and 31 of Table 2, as well as 4A and 4B of Table 3, provide five examples of the conformational descriptor HHBb. This latter shares with HHBh, described previously, the conformation of the first three residues and the sign reversal of the ϕ_*i* + 4_ torsion angle with respect to the preceding residues, but it differs in that residue *i* + 4 belongs to the “b” (bridge, ϕ positive) region of the ϕ,ψ space rather than to the left‐handed helical region “h.” The observed ranges of ϕ,ψ values for residues *i* + 1 to *i* + 3 of the five entries classified as HHBb (ϕ_*i* + 1_ from −75° to −58°, ψ_*i* + 1_ from −44° to −31°; ϕ_*i* + 2_ from −63° to −56°, ψ_*i* + 2_ from −32° to −23°; and ϕ_*i* + 3_ from −113° to −84°, ψ_*i* + 3_ from −9° to 16°) are not too dissimilar from those of the conformational descriptor HHBh. As for residue *i* + 4, the ϕ_*i* + 4_ values range from 92° to 97°, and the ψ_*i* + 4_ values from −4° to 12°. The remarks outlined earlier about the possible occurrence of one or two β‐turns within the π‐turn for the conformational descriptor HHBh apply to type HHBb as well. Indeed, for Entries 9 and 31 of Table 2, a single β‐turn is found at the level of residues *i* + 2 to *i* + 3, whereas Entry 16 of Table 2 and both independent molecules, A and B, of Entry 4 of Table 3 feature two β‐turns.

We identified two examples of π‐turn in linear peptides, which can be described as HHHh (Table 2, Entries 26 and 28). The ϕ,ψ sets for residues *i* + 1 and *i* + 2 are comparable to those found for the conformational descriptor HHBh, whereas those adopted by residue *i* + 3 belong to the helical region according to our classification but are not far from the boundary with region “B,” particularly in terms of the ψ value (Entry 26: ϕ_*i* + 3_,ψ_*i* + 3_ = −79°, –22°; Entry 28: ϕ_*i* + 3_,ψ_*i* + 3_ = −73°, –18°). For both entries, a single β‐turn is found at the level of residues *i* + 2 to *i* + 3, which can be considered as intermediate between Types III and I.

Up to this point, we have described three types of π‐turn characterized by a right‐handed helical conformation for both residues *i* + 1 and *i* + 2 (types HHBh, HHBb, and HHHh). Additional possibilities for the occurrence of a π‐turn emerged from our survey.

Entry 3 of Table 3 provides one example in which residue *i* + 1 is right‐handed helical, both residues *i* + 2 and *i* + 3 belong to the “B” (“bridge,” ϕ negative) region, and residue *i* + 4 is left‐handed helical, thus generating the conformational descriptor HBBh. Two consecutive β‐turns are found within the π‐turn.

In Entry 14 of Table 2, the ϕ,ψ sets for residues *i* + 1, *i* + 2, and *i* + 3 are all located within the “B” region of the conformational space, whereas residue *i* + 4 is left‐handed helical. Therefore, the π‐turn can be defined as type BBBh. A β‐turn (broadly amenable to Type I) occurs at the level of residues *i* + 1 to *i* + 2.

### 3.4 Types hhbH and hhbB

In our notation for the conformational description of π‐turns, the mirror images of types HHBh and HHBb described previously are hhbH and hhbB, in which both residues *i* + 1 and *i* + 2 are left‐handed helical, residue *i* + 3 is found in the “b” (bridge, ϕ positive) region, and residue *i* + 4 is either right‐handed helical or belongs to the “B” (bridge, ϕ negative) region of the ϕ,ψ space. We found four examples of type hhbH π‐turns (Table 2, Entries 2, 3, 19B, and 29) and one example of type hhbB (Table 2, Entry 19A).

Interestingly, among the four examples of type hhbH π‐turns (Table 2, Entries 2, 3, 19B, and 29), the configurations of residues *i* + 1 to *i* + 4 internal to the π‐turn are L‐achiral‐achiral‐L (Entry 2), L‐achiral‐D‐L (Entries 3 and 19B), and D‐achiral‐D‐D (Entry 29). In each of them, one chiral residue adopts the less favored screw sense. This is the case for the left‐handed helical L residue at position *i* + 1 for Entries 2, 3, and 19B, as well as for the right‐handed helical D residue at position *i* + 4 of Entry 29. In Entries 2, 3, and 19B, two consecutive β‐turns of Types III′–I′ are found within the π‐turn, whereas in Entry 29, a single β‐turn (Type I′) is found at the level of residues *i* + 2 to *i* + 3. The left‐handed helical ϕ,ψ values for residue *i* + 1 of Entry 29 (71°, 46°) are too large to allow the onset of a Type‐III′ β‐turn at the level of residues *i* + 1 to *i* + 2. Indeed, the O_*i*_ …. H_*i* + 3_ separation is 2.90 Å, outside the upper limit for the occurrence of a hydrogen bond.

In the crystal structure of Entry 19, Table 2, two independent molecules compose the asymmetric unit. One of them, namely, Entry 19B, featuring a type hhbH π‐turn, is discussed previously. For the other molecule, Entry 19A, we assign the π‐turn to type hhbB. Indeed, the values of backbone torsion angles for residues *i* + 1, *i* + 2, and *i* + 3 closely match (within ±5°) those found for the corresponding residues in Entry 19B. Conversely, the “B” (bridge, ϕ negative) conformation of residue *i* + 4 for Entry 19A, characterized by ϕ_*i* + 4_,ψ_*i* + 4_ = −99°, −11°, differs from the right‐handed helical conformation adopted by residue *i* + 4 for Entry 19B (ϕ_*i* + 4_,ψ_*i* + 4_ = −81°, −30°). In Entry 19A, a single β‐turn (Type I′) internal to the π‐turn is observed at the level of residues *i* + 2 to *i* + 3.

### 3.5 Types PbBb and p*cis*P*cis*P*cis*B

**FIGURE 4:**
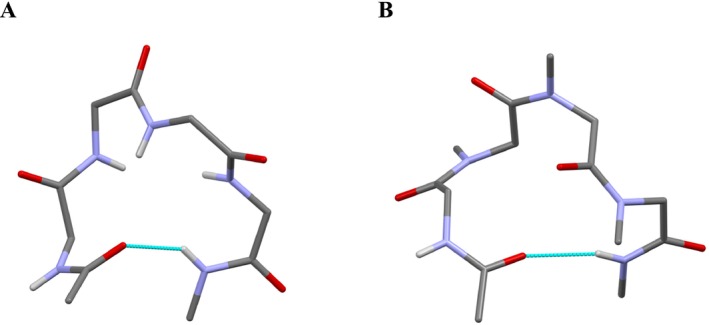
Examples of π‐turns of (A) type PbBb and (B) p*cis*P*cis*P*cis*B. Adapted from [89, 92], respectively. Side chains are omitted for clarity.

A single example of π‐turn characterized by a nonhelical conformation of residue *i* + 1 is offered by Entry 5 of Table 3. This cyclooctapeptide is peculiar in that a second cyclization is provided by a covalent bond between the side chains of residues *i* + 1 and *i* + 5. In terms of the configuration of the residues internal to the π‐turn, L residues occur at positions *i* + 1 and *i* + 3, while residues *i* + 2 and *i* + 4 are achiral. Residue *i* + 1, with ϕ_*i* + 1_,ψ_*i* + 1_ = −75°, 130°, belongs to the “P” (Type‐II polyPro like, ϕ negative) region of the ϕ,ψ map, residues *i* + 2 and *i* + 4 to the “b” (bridge, ϕ positive) region (ϕ_*i* + 2_,ψ_*i* + 2_ = 121°, −22°; ϕ_*i* + 4_,ψ_*i* + 4_ = 132°, −1°), whereas residue *i* + 3 belongs to the “B” (bridge, ϕ negative) region (ϕ_*i* + 3_,ψ_*i* + 3_ = −127°, 14°), thus generating the conformational descriptor PbBb (Figure 4A). No additional intramolecular hydrogen bonds occur within the π‐turn.

Entry 1 of Table 3 remains to be analyzed. The sequence of the four residues internal to the π‐turn is D‐Phe‐L‐Pro‐L‐Pro‐L‐MeAla. Notably, the *cis‐*disposition is observed for the tertiary peptide bonds between D‐Phe and L‐Pro (ω_*i* + 1_ = 4°), between L‐Pro and L‐Pro (ω_*i* + 2_ = −6°), and between L‐Pro and L‐MeAla (ω_*i* + 3_ = 11°). On the basis of the ϕ,ψ sets (ϕ_*i* + 1_,ψ_*i* + 1_ = 89°, −124°; ϕ_*i* + 2_,ψ_*i* + 2_ = −67°, 161°; ϕ_*i* + 3_,ψ_*i* + 3_ = −81°, 160°; and ϕ_*i* + 4_,ψ_*i* + 4_ = −124°, 20°), residue *i* + 1 can be classified as “p” (polyPro like, ϕ positive), residues *i* + 2 and *i* + 3 as “P” (polyPro like, ϕ negative), and residue *i* + 4 as “B” (bridge, ϕ negative). To avoid confusion with the conformational descriptors that we exploited for π‐turns featuring a *trans‐*disposition for all of the ω torsion angles, in this case we thought it useful to place a “*cis*” in between the symbols used to describe the conformation of residues whenever a *cis* ω torsion angle occurs. Therefore, we assign the π‐turn adopted by Entry 1 of Table 3 to type p*cis*P*cis*P*cis*B (Figure 4B). Obviously, no additional intramolecular hydrogen bonds within the π‐turn are possible for this compound.

### 3.6 Overall π‐Turn Types Distribution and Additional Intramolecular Hydrogen Bonds

Table 4 provides a summary of our classification of the 55 occurrences of π‐turns found in the X‐ray diffraction structures of peptides. As many as 78% of the occurrences belong to types HHBh (39 examples) and its mirror image hhbH (four examples). The second more populated mirror‐image pair, accounting for 11% of the occurrences, is represented by types HHBb (five examples) and hhbB (one example). Two examples have been found of type HHHh, whereas a single occurrence is documented for each of the types BBBh, HBBh, PbBb, and p*cis*P*cis*P*cis*B.

**TABLE 4:** Distribution of the occurrence of π‐turns in the X‐ray diffraction structures of peptides among the conformational descriptors exploited in this work.

π‐Turn type	β‐Turn *i* ← *i* + 3	β‐Turn *i* + 1 ← *i* + 4	β‐Turns *i* ← *i* + 3 and *i* + 1 ← *i* + 4	α‐Turn *i* ← *i* + 4	None	π‐Turn type occurrences
HHBh	1	16	20	1	1	39
hhbH		1	3			4
HHBb		2	3			5
hhbB			1			1
HHHh		2				2
BBBh	1					1
HBBh			1			1
PbBb					1	1
p*cis*P*cis*P*cis*B					1	1
Total	2	21	28	1	3	55

*Note:* For each π‐turn type, the number of entries characterized by additional intramolecular hydrogen bonds encompassed within the π‐turn and their location is reported.

Types HHBh/hhbH, HHBb/hhbB, HHHh, BBBh, and HBBh can be considered part of the same family in which all four residues internal to the π‐turn are either helical or belong to the “bridge” region, but residue *i* + 4 is characterized by the reversal of the ϕ sign with respect to the three preceding residues.

A comparison is worth of our results with those reported by Rajashankar and Ramakumar about the occurrence of π‐turns in proteins [40]. By analyzing a set of 228 X‐ray diffraction structures of proteins, they identified 486 examples of π‐turns. About three‐quarters of them are characterized by the screw‐sense reversal of residue *i* + 4 with respect to the preceding residues. Most of them basically correspond to type HHBh in our notation, that is, which largely prevails in our survey on peptides. The second most populated type of π‐turn in proteins, accounting for 22% of the occurrences, is characterized by a right‐handed helical conformation of all residues internal to the turn. We did not find any example of such conformation (it would be type HHHH in our notation) in the crystal structures of peptides.

Concerning the presence of additional hydrogen bonds within the π‐turn, it can be deduced from Table 4 that about one‐half of the π‐turn occurrences contain two consecutive β‐turns, and 38% feature one β‐turn with residues *i* + 2 and *i* + 3 at the corner positions. Conversely, only two examples carrying a single β‐turn at the level of residues *i* + 1 and *i* + 2 have been found, and a single example of an α‐turn within the π‐turn is documented. Our analysis does not support a univocal correlation between the π‐turn type and the pattern of additional hydrogen bonds. Indeed, all possibilities are covered by type HHBh alone. Clearly, the absence or presence and positioning of additional hydrogen bonds within the π‐turn depend on the combination of the specific ϕ,ψ sets adopted by residues *i* + 1, *i* + 2, and *i* + 3, whereas the conformation of residue *i* + 4 is not relevant, because the latter may only provide one of the N–H donors. Therefore, the two mirror‐image pairs HHBh/hhbH and HHBb/hhbB can be considered as equivalent to each other in terms of additional hydrogen‐bonding potential. All of the π‐turn types HHHh, BBBh, and HBBh, even though their population is limited to one or two occurrences, provide examples of the presence of one (either *i* ← *i* + 3 or *i* + 1 ← *i* + 4) or two β‐turns within the π‐turn. Conversely, types PbBb and p*cis*P*cis*P*cis*B (one example each) are devoid of additional hydrogen bonds. Whereas the occurrence of β‐turns within a type p*cis*P*cis*P*cis*B π‐turn is clearly impossible, it has to be noted that, in principle, type PbBb may be compatible with the onset of a Type‐II β‐turn at the level of residues *i* + 1 and *i* + 2, provided that their ϕ,ψ sets would be sufficiently close to the ideal values for such folded conformation (ϕ_*i* + 1_,ψ_*i* + 1_ = −60°, 120°; ϕ_*i* + 2_,ψ_*i* + 2_ = 80°, 0°) [6], which is not the case for Entry 5 of Table 3.

No matter which additional hydrogen bonds occur within a π‐turn (*i* ← *i* + 3, *i* + 1 ← *i* + 4, or *i* ← *i* + 4, see Table 4 and Figure 3), the N–H groups of residues *i* + 1 and *i* + 2 remain free from hydrogen bonding internal to the π‐turn. Therefore, in linear peptides, they are potentially available for hydrogen bonding to C=O groups upstream in the peptide chain, compatibly with the main‐chain length. The same may also hold true for the N–H group of residue *i* + 3, unless this latter is already engaged in an *i* ← *i* + 3 β‐turn within the π‐turn, as it happens for entries marked with note “a” in Table 2.

In general, when the backbone preceding the π‐turn is helical with an exclusive or prevailing α‐helical character, the N–H groups of at least residues *i* + 1 and *i* + 2 (Table 2, Entries 16, 18, 19B, and 24–27) or even the N–H groups of all residues *i* + 1 to *i* + 3 (Table 2, Entries 17, 19A, 21A, 21B, 23, and 28–30) are involved as H‐bond donors of α‐turns. Conversely, the N–H groups of residues *i* + 1 and *i* + 2 are part of (usually Type III) β‐turns in the case of fully developed or incipient 3_10_‐helices (Table 2, Entries 6A, 7, 8, and 13–15).

There are also examples in which the N–H group of residue *i* + 1 is involved in a β‐turn while those of residues *i* + 2 and *i* + 3 are H‐bond donors of α‐turns. Such arrangement is observed for a number of hepta‐ and octapeptides (Table 2, Entries 4, 5, 6B, 9, and 11), thus giving rise to an incipient helix with a mixed 3_10_/α‐character preceding the π‐turn. The same situation is found in the case of two longer peptides (Table 2, Entries 22 and 31) in which the π‐turn is not located at the C‐terminus of the peptide chain. Specifically, in the decapeptide Boc‐L‐Leu‐Aib‐L‐Val‐Aib‐L‐Leu‐Aib‐L‐Val‐D‐Ala‐D‐Leu‐Aib‐OMe (Table 2, Entry 22), the π‐turn encompasses Residues 3–6. It is preceded at the N‐terminus by a right‐handed, incipient 3_10_‐/α‐helix, and it is followed at the C‐terminus by a left‐handed 3_10_‐helix. Similarly, in the 17‐mer Boc‐L‐Val‐L‐Ala‐L‐Leu‐Aib‐L‐Val‐L‐Ala‐L‐Leu‐Gly‐Gly‐L‐Leu‐L‐Phe‐L‐Val‐D‐Pro‐Gly‐L‐Leu‐L‐Phe‐L‐Val‐OMe (Table 2, Entry 31), Residues 5–8 are internal to the π‐turn. The conformations of Residues 1 and 2 are extended and *semi*‐extended, respectively. They are followed by an incipient 3_10_‐/α‐helix generated by the succession of one Type‐III β‐turn and two α‐turns, the H‐bond donors of which are the N–H groups of Residues 5–7 (i.e., those occupying positions *i* + 1 to *i* + 3 within the π‐turn). Residues 9–17 give rise to a β‐hairpin [two antiparallel β‐strands connected by a Type‐II′ β‐turn with D‐Pro (13) and Gly (14) as corner residues].

Among the linear peptides listed in Table 2, the lack of intramolecular H‐bonds involving the N–H groups of residues *i* + 1 and/or *i* + 2 internal to the π‐turn is obviously observed not only for Entry 2, a pentapeptide in which there are no H‐bond acceptors upstream to the π‐turn, but also in the case of Hexapeptide 3, in which the possible formation of an intramolecular H‐bond between the N–H group of residue *i* + 2 and the urethane carbonyl oxygen of the N‐terminal Boc group is hampered by the right‐handed helical conformation adopted by L‐Leu (1), preceding a π‐turn of type hhbH, thus characterized by a left‐handed helical conformation of residue *i* + 1.

**FIGURE 5:**
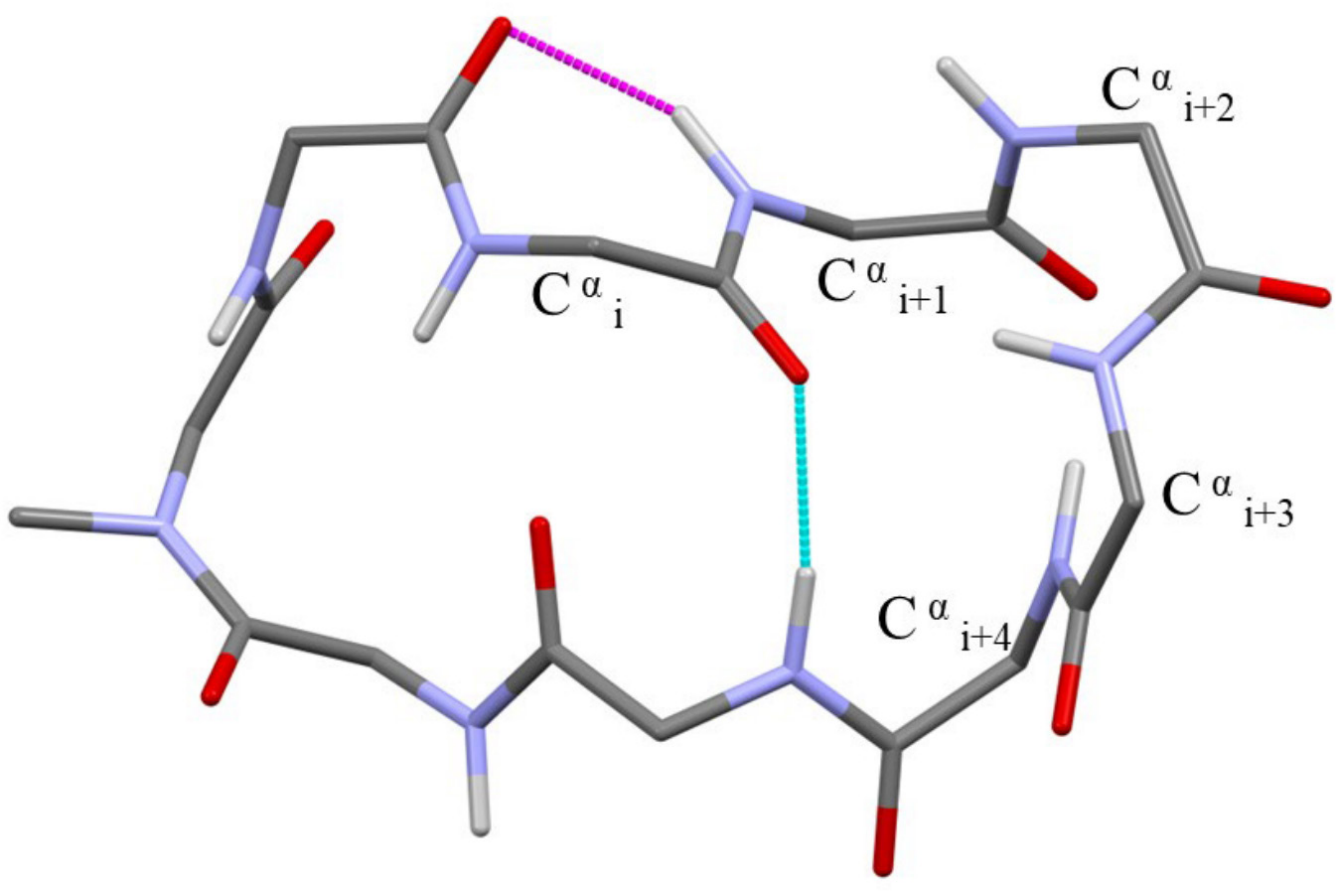
Example of a γ‐turn (H‐bond in magenta) preceding a π‐turn in the structure of a cyclononapeptide. Adapted from [101]. Side chains are omitted for clarity.

Concerning cyclopeptides, among the 17 occurrences of π‐turns listed in Table 3, there are 12 examples of type HHBh (Entries 2A, 2B, and 6–15), two of type HHBb (Entries 4A and 4B), and one of type HBBh (Entry 3). All of them contain two consecutive β‐turns, stabilized respectively by *i* ← *i* + 3 and *i* + 1 ← *i* + 4 hydrogen bonds, within the π‐turn. The N–H groups of residues *i* + 1 and *i* + 2 are usually involved in *intermolecular* hydrogen bonds. However, in a not negligible number of cases (Entries 6, 8–11, and 13–15) the N–H group of residue *i* + 1 is *intramolecularly* hydrogen bonded to the carbonyl oxygen atom of residue *i* − 1 (the residue preceding that, which acts as the hydrogen bond acceptor for the π‐turn), thus giving rise to a γ‐turn centered on residue *i* (Figure 5). In these eight cases, the observed ϕ,ψ values for residue *i* are in the ranges of −94° ≤ ϕ_*i*_ ≤ −84° and 56° ≤ ψ_*i*_ ≤ 64°. Negative ϕ_*i*_ and positive ψ_*i*_ values, but larger in magnitude, characterize most of the remaining cyclopeptides listed in Table 3, including Entries 2A, 2B, and 3, which lack the potential N–H hydrogen‐bonding donor at residue *i* + 1. In particular, the ϕ_*i*_,ψ_*i*_ sets *i* of Entries 4A and 4B are not far from those of the “ideal” γ‐turn (−75°, 65°), but the relevant H…O separations are in the range of 2.57–2.73 Å, thus exceeding the commonly accepted limit of 2.50 Å for the occurrence of a H‐bond. On these bases, the conformation of residue *i* of Entries 4A and 4B can be described as an “open” γ‐turn.

### 3.7 Screw‐Sense Reversal of Residue *i* + 4

In proteins, the screw‐sense reversal of residue *i* + 4 typical of π‐turns acting as the helix C‐terminating Schellman motif is usually performed by the achiral Gly residue [49, 50, 51, 52, 53, 54, 55]. Conversely, among the linear peptides featuring one of the π‐turn types characterized by screw‐sense reversal (HHBh/hhbH, HHBb/hhbB, HHHh, BBBh, and HBBh), position *i* + 4 is occupied by Gly only once (Table 2, Entry 31). In most cases, the screw‐sense reversal is associated with the presence of a *noncoded*, achiral α‐amino acid residue at position *i* + 4, which results from the sequence design of these synthetic peptides. Specifically, the purpose of the screw‐sense reversal is accomplished by the C^α^‐tetrasubstituted Aib residue in 20 occurrences (Entries 1, 4–8, 10–13, 22, 24–27, and 30) and by the C^α^,C^β^‐didehydro α‐amino acid residue ΔPhe in three examples (Entries 9, 14, and 15). There are also eight examples in which the left‐handed screw sense (opposite to that of the preceding residues) at position *i* + 4 is adopted by a protein amino acid residue of D configuration (Entries 16, 17A, 17B, 18, 20, 21A, 21B, and 23) and four examples of a protein amino acid residue of L configuration of right‐handed screw sense (opposite to that of the preceding residues) at position *i* + 4 (Entries 2, 3, 19A, and 19B). In only one case, namely, Entry 29, in which the configurations of residues *i* + 1 to *i* + 4 internal to the π‐turn are D‐achiral‐D‐D, the screw‐sense reversal at position *i* + 4 is performed by a right‐handed D‐Leu.

On the other hand, cyclopeptides listed in Table 3 are natural compounds or analogues thereof. They provide 15 occurrences of the π‐turn types HHBh, HBBh, or HHBb characterized by a left‐handed conformation of residue *i* + 4. Such screw‐sense reversal is provided by Gly in two cases (Table 3, Entries 2A and 2B) and by a protein amino acid residue of D configuration in six cases (Entries 3, 4A, 4B, 10, 11, and 15). However, there are as many as seven examples in which the screw‐sense reversal at position *i* + 4 is performed by a protein amino acid residue of L configuration (Entries 6–9 and 12–14). This latter finding should not be overemphasized because our dataset of cyclopeptides is rather limited in size and molecular diversity, as well as to some extent biased by the occurrence of solvatomorphs of the same compound. In any case, it suggests that in cyclononapeptides, the energy penalty associated with an L‐amino acid adopting the less favored left‐handed helical screw sense is somehow compensated for in the frame of a π‐turn.

## 4 Conclusions

The set of 55 occurrences of π‐turns in the crystal structures of peptides covered by our survey has significantly expanded, both in size and in diversity, compared to that reviewed by Rajashankar and Ramakumar in 1996 [40]. At that time, only nine examples of π‐turns in linear peptides were available, all of them positioned at the C‐end of a helix and characterized by the screw‐sense reversal of residue *i* + 4 with respect to the preceding ones.

Our analysis shows that π‐turns featuring a helical conformation for residues *i* + 1 and *i* + 2, a conformation falling in the “bridge” region of the Ramachandran map for residue *i* + 3, and a helical conformation for residue *i* + 4 but with a screw sense opposite to that of the three preceding residues (types HHBh and its mirror image hhbH in our notation) are by far the most abundant types of π‐turn in our dataset. Occasionally, the helical conformation of one or two amino acid residues can be replaced by a “bridge” conformation or vice versa, while keeping the screw‐sense reversal for residue *i* + 4.

When a type HHBh π‐turn acts as a C‐capping motif of a helix, residues *i* + 1 and *i* + 2 internal to the π‐turn are also part of the preceding helix, suggesting that the onset of the π‐turn may somehow benefit from the helical pre‐organization of residues *i* + 1 and *i* + 2. However, a π‐turn of this type (or of the related types HBBh and HHBb) occurs not only at the C‐end of fully developed or incipient α‐helices, 3_10_‐helices, and mixed α‐/3_10_‐helices, but also it can even stand on its own, without the support of a preceding helix, as shown by two structures of linear peptides and, most notably, by 15 examples of cycloocta‐ and cyclononapeptides.

The presence of additional intramolecular H‐bonds internal to π‐turn types characterized by screw‐sense reversal of residue *i* + 4 seems to be the rule rather than the exception. Specifically, in about one‐half of the cases, two consecutive (*i* ← *i* + 3 and *i* + 1 ← *i* + 4) β‐turns are found, and in more than one‐third, a single, *i* + 1 ← *i* + 4, β‐turn is observed. The occurrence of an *i* ← *i* + 3 β‐turn or of an *i* ← *i* + 4 α‐turn is also documented. Additional hydrogen bonds within the π‐turn clearly provide a significant contribution to the energetic stabilization of this folded conformation. In this connection, the possibility offered by types HHBh, HBBh and HHBb π‐turns to host up to two additional intramolecular hydrogen bonds is unique among the classes of intramolecularly hydrogen‐bonded turns.

Cyclopeptides offered examples of two types of π‐turn that, to the best of our knowledge, have no parallel in the structures of proteins. One of them is characterized by the *cis‐*disposition of three tertiary peptide bonds. The second π‐turn type features residue *i* + 1 in a Type‐II polyPro‐like conformation, followed by three residues, all of them in the “bridge” conformation but with their ϕ torsion angles alternating in sign.

In proteins, π‐turns characterized by helical ϕ,ψ sets of the same screw sense for all internal residues are well documented. When positioned within an α‐helix, they are responsible for the onset of the so‐called π‐bulges [36, 40, 41, 42, 43, 44]. They are also compatible with the occurrence of multiple, consecutive π‐turns. Indeed, stretches of rather irregular π‐helices up to 13 residues in length have been found [42]. Conversely, in the crystal structures of peptides, π‐turns of such type are hitherto unreported, and, as a consequence, it comes without surprise that a regular π‐helix still awaits authentication at atomic resolution.

In a significant number of the π‐turns characterized by the screw‐sense reversal of residue *i* + 4 with respect to the preceding residues, in linear peptides, this position is occupied by the strongly helicogenic, achiral, C^α^‐tetrasubstituted Aib residue [103]. A π‐turn of this type might be of interest as a helical C‐capping motif for the development of peptides and peptidomimetics able to interfere with protein–protein interactions between portions of α‐helical surfaces that are involved in cell signaling pathways [104, 105, 106, 107, 108, 109, 110, 111]. To this purpose, a C‐capping motif should ideally combine its role as a helix terminator with some sort of helix‐stabilizing effect in the preceding, usually relatively short, peptide segment. Therefore, an Aib residue at position *i* + 4 seems more appropriate than the achiral but conformationally flexible Gly residue usually found at the same position in the Schellman motifs of proteins. However, to ensure the screw‐sense reversal, a chiral residue would be preferable. In our opinion, for the rational design of a π‐turn characterized by the screw‐sense reversal of residue *i* + 4 with respect to the preceding residues in linear or cyclic peptides/peptidomimetics, replacement of Aib by C^α^‐methylvaline [(αMe)Val] might be rewarding. Indeed, among the C^α^‐methylated analogues of protein amino acids, in addition to being restricted to the helical region of the ϕ,ψ space, (αMe)Val is endowed with the strongest bias for a single helical screw sense (i.e., left handed for the D enantiomer) [112].

## Conflicts of Interest

The authors declare no conflicts of interest.

## Data Availability

The data that support the findings of this study are available upon request from the corresponding author. The data are not publicly available because of privacy or ethical restrictions.
